# Deciphering HER2 Breast Cancer Disease: Biological and Clinical Implications

**DOI:** 10.3389/fonc.2019.01124

**Published:** 2019-10-29

**Authors:** Ana Godoy-Ortiz, Alfonso Sanchez-Muñoz, Maria Rosario Chica Parrado, Martina Álvarez, Nuria Ribelles, Antonio Rueda Dominguez, Emilio Alba

**Affiliations:** ^1^Unidad de Gestión Clínica Intercentros de Oncología Medica, Hospitales Universitarios Regional y Virgen de la Victoria de Málaga, Málaga, Spain; ^2^Laboratorio de Biología Molecular del Centro de Investigaciones Médico-Sanitarias de Málaga (CIMES), Instituto de Investigación Biomédica de Málaga (IBIMA), Universidad de Málaga (UMA), Málaga, Spain; ^3^Centro de Investigación Biomédica en Red de Oncología, CIBERONC-ISCIII, Madrid, Spain

**Keywords:** breast cancer, HER2-positive, intrinsic subtype, heterogeneity, HER2-enriched, molecular

## Abstract

The main obstacle for designing effective treatment approaches in breast cancer is the extensive and the characteristic heterogeneity of this tumor. The vast majority of critical genomic changes occurs during breast cancer progression, creating a significant variability within primary tumors as well as between the primary breast cancer and their metastases, a hypothesis have already demonstrated in retrospective studies (1). A clear example of this is the HER2-positive breast cancer. In these tumors, we can find all of the transcriptional subtypes of breast cancer, even the basal like or luminal A subtypes. Although the HER2-enriched is the most representative transcriptional subtype in the HER2-positive breast cancer, we can find it too in breast cancers with HER2-negative status. This intrinsic subtype shows a high expression of the HER2 and is associated with proliferation-related genes clusters, among other features. Therefore, two hypotheses can be suggested. First, the HER2 amplification can be a well-defined driver event present in all of the intrinsic subtypes, and not a subtype marker isolated. Secondly, HER2-enriched subtype can have a distinctive transcriptional landscape independent of HER2 amplification. In this review, we present an extensive revision about the last highlights and advances in clinical and genomic settings of the HER2-positive breast cancer and the HER2-enriched subtype, in an attempt to improving the knowledge of the underlying biology of both entities and to explaining the intrinsic heterogeneity of HER2-positive breast cancers.

## Introduction

Breast cancer (BC) is the most common malignant tumor in women and one of the principal causes of cancer mortality in this sex, despite significant improvements obtained in the lasts decades. Conversely, male breast cancer is a rare disease with an incidence of <1% and mainly classified by immunohistochemistry as a luminal disease (2). BC is modeled by a group of heterogeneous diseases, at both an inter- and intra-tumoral level. All of them share a substantial morphological and molecular heterogeneity, what affect to his clinical behavior and therapeutic response. A crucial objective in the treatment of any cancer disease is to perform clinical decisions through a comprehensive insight of the molecular profile of the tumor to predict the probable clinical outcome of the disease individually. By the expansion of high-throughput molecular technologies, we can analyze changes in the genetic, epigenetic and proteomics contexts, so that allows improving in the comprehension of the complexity of BC biology.

One biomarker with reported heterogeneity in BC is the *Human Epidermal Growth Factor Receptor 2* (HER2), a component of the *EGF* receptor (EGFR) family. The overexpression of this biomarker defined the HER2-positive disease. Traditionally, HER2-positive breast cancer (HER2+ BC) has been associated with a worse prognosis and inferior outcomes in survival. However, over the last years, several therapeutic advances have been improved the clinical treatment of HER2+ disease, and thus, its prognosis. After the discovery of the intrinsic subtypes through gene expression analysis, and later transcriptomic and genomic studies, there is sufficient evidence that HER2+ BC is an entity with a large heterogeneity at multiple levels (3), including cell-to-cell. There has been discrepancy about the determination of the clinical status of HER2+ over the last years, with several guidelines and updates in order to find a formal and universal consensus. In clinical practice, HER2+ tumors are categorized by immunohistochemistry (IHC) and/or by *in situ hybridization* (ISH) in order to tailor the different therapeutic approaches (4).

The gene expression profiling has had a large-scale impact in the progress about the knowledge of the biological heterogeneity of this tumor (5). However, in this ambit, there is a considerable variability as well, what makes it even more difficult to categorize the basis of pathological diagnosis and therapeutic approach. The principal molecular subtypes of BC have widely characterized, and within HER2+ BC the most representative intrinsic subtype is the HER2-enriched (HER2-E). However, we can find HER2+ BC with luminal A, luminal B, or even the basal-like subtype (6). The intrinsic subtype HER2-E is defined generally by a higher expression of HER2 at the RNA and protein level than other subtypes, in addition the increased expression of the tumor proliferation-related genes (6, 7). Recent studies confirm that this subtype obtains the best clinical and therapeutic results by anti-HER2 therapies, with or without chemotherapy, in both adjuvant and neoadjuvant scenarios, and regardless of the clinical status of HER2 (3). Nonetheless, no more than 50% of clinically HER2+ tumors are HER2-E, and what is more exciting, we can also find this subtype in clinically HER2-negative BC, which do not receive HER2-therapies since these drugs are not approved for the treatment of clinically HER2-negative breast tumors. Therefore, we consider it is highly important to perform an extensive revision about the latest highlights and advances in clinical outcomes and genomic features within HER2+ BC and its most representative intrinsic subtype, HER2-E, with a previous extensive revision from the state of science in which these advances are based.

## Current Classification of Breast Cancer

Intertumoral heterogeneity of BC is initially illustrated with a clinical staging of the disease. The TNM staging system by the *American Joint Committee on Cancer and Union for International Cancer Control (AJCC/UICC)* adds information about tumor features such as size, regional lymph-node involvement or the presence of distant metastases (8). After the clinical diagnosis, the first step is the assessment of histological criteria on the primary tumor obtained by surgery and/or a core biopsy, encompassing morphology-base and immunohistochemical (IHC) analyses for testing the biomarker profile. This is a classical and non-molecular classification of BC, and sets the standard in the usual clinical practice. Classic pathological criteria, such as histological type, tumor size, grade and axillary lymph node status, are relevant for the initial prognostic evaluation (9). The expression of hormone receptors [estrogen (ER) and progesterone receptors (PR)] by IHC and the overexpression and/or amplification of HER2 by IHC and/or ISH gives additional predictive value, being elementary for guiding algorithms of treatment (9, 10), as will be discussed in the following two sections.

### Histopathological Subtypes: Morphologic Heterogeneity

The histopathological classification of BC is set by the 2012 *World Health Organiza*tion (WHO) (11). Most of the breast cancers are adenocarcinomas, with around 70–80% defined as invasive ductal carcinomas not otherwise specified (IDC-NOS) (11). The rest, around 25–30%, are characterized by “*histological special types”* such as papilar, metaplastic, cribiform, apocrine, or mucinous carcinomas, among others (11). The majority of special types is rare and differ strongly about prognosis and response to the treatments (12). The tumor grade is the other important intrinsic characteristic of tumoral heterogeneity (13, 14).

### Immunohistochemistry: ER, PR, and HER2

Via the characterization of ER, PR, and HER2 status, we can divide BC in three phenotypes or entities. Hormone receptor-positive breast cancers are defined as positive by expression of ER and/or PR receptor equal to 1% or higher of invasive cancer cells (15). ER and PR receptors are expressed around 80 and 65% of breast cancers, respectively (16). Although estrogen receptor-positive tumors co-express PR in the majority of breast cancers, some cases are ER+/PR– and less frequently, ER–/PR+. The response to hormonal therapy seems to be major in breast tumors with positivity for ER and PR, with lower rates in ER+/PR– and ER–/PR+ tumors (11).

Approximately 15–20% of BC has HER2 overexpression and/or amplification, and over 50% of these co-expressing hormone receptors (13, 17). These tumors are called HER2+ BC. The remaining, with negativity for hormonal receptors and HER2, are denominated triple-negative breast cancers. A fourth protein marker, the androgen receptor (AR), is immunoexpressed in 60–80% of breast cancers, with similar proportions to prostate tumors, and specially expressed in HER2+ and triple-negative breast tumors. However, its determination is still not justified in clinical practice as there is no targeted treatment approved for this marker. Other biomarkers with heterogeneous expression include the *epidermal growth factor receptor* (EGFR), p53, c-myc, and proliferation markers such as Ki-67 (14, 18). Ki-67 is a nuclear protein, expressed in all phases of the cell cycle except G0, and a cellular marker of proliferation with prognostic and predictive value (16, 19).

Even so, this current and basic classification of human breast tumors presents a number of important limitations. The main one is the variability in therapeutic response and clinical outcomes, even for tumors with similar clinical and pathological features. Secondly, this classification provides limited knowledge into the biology and the molecular pathways that divide the BC in distinct subtypes and stages, stepping away from the personalized treatment paradigm.

### Molecular and Genomic Classification of Breast Cancer

Expression analysis has provided an opportunity to explore comprehensive molecular profiling of BC. Differences in gene expressions patterns display basic alterations in the tumor cell biology and are associated with significant variation in terms of clinical behavior, survival (17, 20–22), and treatment outcomes (23–37). The identification of several molecular subtypes was the first insight into the molecular heterogeneity of the BC (20). Five main intrinsic subtypes have been identified based solely on gene expression patterns using DNA microarrays (20, 22): *luminal A, luminal B*, HER2 overexpressing or *HER2-enriched* (HER2-E) and *basal like*, with another less characterized group named *normal breast-like*. They are called as “intrinsic subtypes of breast cancer” and they have exposed crucial differences in several aspects. The tumor heterogeneity within hormone receptor-positive breast cancers are encompassed by the *luminal A* and *luminal B* subtypes, with better survival outcomes with respect to the non-luminal intrinsic subtypes. The *luminal B* breast tumor expresses hormonal receptors same as the luminal A subtype, but generally having low PR, high proliferation, high grade and worse response to hormonal therapy. At the molecular level, this subtype seems to be dramatically distinct from luminal A, at levels of gene expression, gene copy, or somatic aberrations. All of these features, confers it worse prognosis than the other luminal intrinsic subtype (5).

In 2009, Parker et al. (25) introduced a gene expression-based test named PAM50, which identifies the intrinsic molecular subtypes in four well-established transcriptional subtypes, through the expression of 50 genes in formalin-fixed paraffin embedded (FFPE) tumor tissues: *luminal A, luminal B, basal-like, and HER2-enriched* (25, 28, 31). The intrinsic subtypes overlap with staining of ER, PR and HER2 protein expression by IHC and complemented with ISH for testing HER2 gene amplification. However, several studies have assessed and compared the classification of breast tumors based on the PAM50 gene expression with the classification based on pathological criteria, and a low concordance rate was found in the majority of these studies (31, 34–42). For example, in a combined analysis of data from several studies including a total of 5,994 independent tumor samples, the discordance rate was found to be present in 30.72% across all patients (43). The majority of these studies performed central assessment of pathology-based biomarkers, which normally shows less discrepancies than local determination (15). Therefore, the two methods should never be considered the same to identify intrinsic biology of BC.

**Figure 1:**
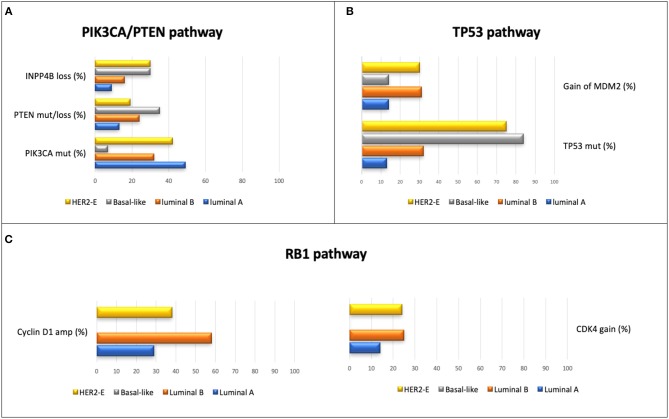
Principal alterations in most representative pathways in BC, according the intrinsic profiling, analyzed in the TGCA project (5); *percentages are based on 466 tumor samples (463 patients)*. **(A)** Principal alterations in TP53 pathway. **(B)** Principal alterations in PIK3CA/PTEN pathway. **(C)** principal alterations in RB1 pathway. Within the basal-like intrinsic subtype, the main alterations found in this pathway were RB1 mut/loss (20%) and amplification of Cyclin E1 (9%). The expression degree of CDKN2C and RB1 was low and high, respectively, in the luminal A subtype, unlike what was reported in tumors with basal-like subtype.

**Figure 2:**
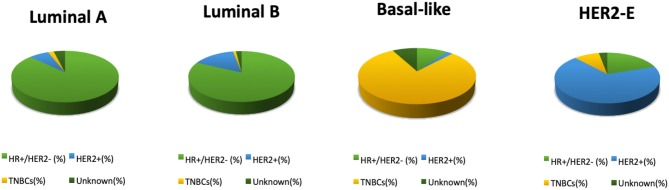
Distribution of PAM50 intrinsic subtypes within each IHC subtype of the breast cancers analyzed in the TGCA project (5); *percentages are based on 466 tumor samples (463 patients)*.

Nonetheless, the diverse genomic landscape of BC is not completely captured through histopathological or transcriptomic analysis. Changes in gene expression patterns are influenced by the underlying genomic structure, and we have evidence that some features of the intrinsic subtypes can be defined by copy number profiling (5, 29, 44) The development of next-generation sequencing technologies has allowed for the characterization of the mutational landscape of this disease, with the identification of novel cancer genes that found it to be recurrently mutated in BC (6, 36, 45, 46). The relevant of integration of the intrinsic subtype with genomic analysis are highlighted in one of the most complete and important molecular characterization studies that have ever been performed in BC (5). In this study, led by *The Cancer Genome Atlas Project* (TCGA), more than 600 primary tumors were extensively profiling at the DNA (methylation, copy-number alterations, somatic and germline mutations), RNA (i.e., miRNA sequencing and mRNA expression) and protein levels (5) (Table 1; Figure 1). After the analysis of more than 300 primary tumors, five different data-types were mixed together in a cluster of 10 clusters. The consensus clustering analysis identified four major groups of BC, which were found to be very-well summarize by the four molecular intrinsic subtypes defined by mRNA expression only (47) (Figure 2).

**Table 1:** Main data about mRNA expression, copy number, DNA mutations and protein expression in the breast cancer tissue samples analyzed in the TGCA project (5).

**Subtype/cluster**	**Luminal A**	**Luminal B**	**Basal-like**	**HER2-E**
mRNA expression	High ER cluster; low proliferation signature	Lower ER cluster; high proliferation signature	Basal-signature; high proliferation	HER2 amplicon signature; high proliferation
Copy number	Most diploid; many with quiet genomes; 1q, 8q, 8q11 gain; 8o, 16q loss, 11q13.3 amp (24%)	Most aneuploidy; many with focal amp; 1q, 8q, 8p11 gain; 8p, 16q loss, 11q13.3 amp (51%); 8p11.23 amp (28%)	Most aneuploidy; high genomic instability; 1q, 10p gain; 8p, 5q loss; MYC focal gain (40%)	Most aneuploidy; high genomic instability; 1q, 8q gain; 80 lossM 17q12 focal ERRB2 amp (71%)
DNA mutations	PIK3CA (49%); TP53 (12%), GATA3 (14%), MAP3K1 (14%)	TP53 (32%); PIK3CA (32%); MAP3K1 (5%)	TP53 (84%); PIK3CA (7%)	TP53 (75%); PIK3CA (42%); PIK3R1 (8%)
Protein expression	High estrogen signaling; high MYB; RPPA reactive subtypes	Less estrogen signaling; high POXM1 and MYC; RPPA reactive subtypes	High expression of DNA repair proteins, PTEN and INPP4B loss signature (pAKT)	High protein and phosphoprotein expression of EGFR and HER2

*Amp, amplification; mut, mutation. Percentages are based on 466 tumor samples (463 patients)*.

Thus, all breast cancers show significant genetic diversity. Inherited variants, represented by the single-nucleotide polymorphisms (SNPs) and copy number variants (CNVs), can have an impact in a germline genetic landscape of the individual and inducing the cancer development. The single-nucleotide variants (mutations) and copy number aberrations (CNAs) are genomic changes at somatic level, thus variations acquired that contribute to the initiation and the dissemination of sporadic breast tumors (48). In a recent study, the authors integrated analysis of both, genomic and transcriptomic data, in 2,000 breast tumors as part of the METABRIC consortium (36) dataset, and proposed an alternative molecular classification (48) (Table 2). Germline variants and somatic alterations were found to be linked with changes in gene expressions, and the CNAs reported the greatest variability. Clustering analysis of joint copy number and gene expression data from the cis-associated gene reported 10 new molecular subgroups or *integrative clusters* with the capacity of dividing the main intrinsic subtypes into independent groups. Each integrative clusters are characterized by distinct CNAs, gene expression changes, clinical characteristics and different survival outcomes (48). This extensive heterogeneity, as a result of different cell-of-origins and molecular variations, makes that the response of patients to treatments remained variable and difficult to predict.

**Table 2:** Main features of the integrative clusters (48) in 2,000 breast tumors samples.

**IntClust**	**Frequency (*n*)**	**Expression (*n*, %)**	**Molecular features**	**PAM50 subtype (*n*, %)**	**Prognosis (HR 5, 10 year DSS)**
1	139	ER+: 123 (88.5%) PR+: 60 (43%) HER2+: 20 (14.4%)	17q23 amplification High genomic instability	Basal: 9 (6.5%) HER2-E: 21 (15 %) LumA: 11 (7.9%) LumB: 90 (64.8%) Normal: 8 (5.8%)	Intermediate 0.80, 0.69
2	72	ER+: 69 (95.8%) PR+: 51 (70.8%) HER2+: 3 (4.2%)	11q13/14 amplificacion High genomic instability	Basal: 2 (2.8%) HER2-E: 6 (8.3 %) LumA: 25 (34.7%) LumB: 36 (50%) Normal: 3 (4.2%)	Poor 0.78,0.51
3	290	ER+: 278 (95.9%) PR+: 211 (72.8%) HER2+: 1 (0.3%)	Paucity of copy number changes Low genomic instability	Basal: 4 (1.4%) HER2-E: 9 (3.1 %) LumA: 195 (67.9%) LumB: 43 (15%) Normal: 36 (12.5%)	Good 0.93, 0.88
4	343	ER+: 238 (69.4%) PR+: 155 (45.2%) HER2+: 20 (5.8%)	CNA devoid Low genomic instability	Basal: 64 (18.7%) HER2-E: 34 (10 %) LumA: 106 (31%) LumB: 29 (8.5%) Normal: 109 (31.9%)	Good 0.89, 0.76
5	190	ER+: 79 (41.6%) PR+: 40 (21.1%) HER2+: 181 (14.4%)	ERBB2 amplification Intermediate genomic instability	Basal: 21 (11%) HER2-E: 108 (56.8 %) LumA: 18 (9.5%) LumB: 33 (17.4%) Normal: 10 (5.3%)	Poor 0.62, 0.45
6	85	ER+: 123 (88.5%) PR+: 60 (43%) HER2+: 20 (14.4%)	8p12 amplificacion High genomic instability	Basal: 3 (3.5%) HER2-E: 10 (11.8%) LumA: 23 (27.1%) LumB: 43 (50.6%) Normal: 6 (7.1%)	Intermediate 0.83, 0.59
7	190	ER+: 187 (98.4%) PR+: 150 (79%) HER2+: 2 (1.1%)	16p gain, 16q loss, 8q amplificacion Intermediate genomic instability	Basal: 3 (1.6%) HER2-E: 9 (4.8 %) LumA: 123 (65.1%) LumB: 41 (21.7%) Normal: 13 (6.9%)	Good 0.94, 0.81
8	299	ER+: 297 (99%) PR+: 236 (78.9%) HER2+: 1 (0.3%)	1q gain, 16q loss Intermediate genomic instability	Basal: 1 (0.3%) HER2-E: 9 (3%) LumA: 192 (64.2%) LumB: 89 (29.8%) Normal: 8 (2.7%)	Good 0.88, 0.78
9	146	ER+: 125 (85.6%) PR+: 79 (54.1%) HER2+: 10 (6.9%)	8q gain, 20q amplificacion High genomic instability	Basal: 20 (13.8%) HER2-E: 26 (18%) LumA: 24 (16.6%) LumB: 70 (48.3%) Normal: 5 (3.5%)	Intermediate 0.78, 0.62
10	226	ER+: 25 (11.1%) PR+: 19 (8.4%) HER2+: 6 (2.7%)	5q loss, 8q gain, 10p gain, 12 p gain Intermediate genomic instability	Basal: 202 (89.4%) HER2-E: 8 (3.5%) LumA: 1 (0.4%) LumB: 14 (6.2%) Normal: 1 (0.4%)	Poor 0.71, 0.68

*IntClust, integrative cluster; DSS, disease-specific survival; ER+, estrogen receptor; PR+, progesterone receptor*.

## HER2-positive Breast Cancer and HER2-enriched Subtype

A clear example of complex heterogeneity, inter- and intratumoral, is the HER2+ BC. ERBB2/HER2 is an oncogene coding for a tyrosine kinase receptor that activates oncogenic pathways related with increase proliferation, angiogenesis and invasiveness, resulting in an highly aggressive neoplasm with poor outcomes that others BC (49, 50). The ERRBB2/HER2 gene is located in chromosomal region 17q12-21 and its amplification occurs in around 15–20% of breast cancers (10). Overexpression of the protein kinase receptor enables patients with HER2+ BC to benefit from antibody-based and anti-kinase based therapies that target this receptor, either with a combination of these targeted therapies and chemotherapy, or through dual anti-HER2 therapy without chemotherapy (51–69). This therapeutic approach, has completely changed the prognosis of HER2+ tumors.

So far, the HER2+ BC has been considered as a simple entity. Although the HER2 receptor itself has a dominant role, and the efficacy of the anti-HER2 agents support it, it is increasing the evidence that HER2 is a phenotype with one of the most extensive and specific heterogeneity (4–6, 70). HER2+ breast cancers vary clearly in their genome variations, gene expression programs, cell-of-origin and cell plasticity, what impact in their microenvironment, prognosis and therapeutic outcomes.

### Immunohistochemistry Criteria: Past, Present, and Future

The HER2 status assessment was establishment by The *American Society of Clinical Oncology* and the *College of American Pathologists (ASCO/CAP)*, with the publication of guidelines with recommendations for testing the level of HER2 protein overexpression by IHC and the HER2 gene amplification determined by ISH, both on FFPE breast tumor tissues. The first ASCO/CAP guideline was published in 2007 (71), and updated in 2013 (72, 73) and 2018 (4) (Table 3). In the last update, the experts refined some controversial criteria of the older guidelines and tried to systematize the testing algorithm for the unusual categories of HER2 ISH results (4) (Table 4). The results of these tests are graded semi-quantitatively as either 0 (negative), 1+ (negative), 2+ (equivocal) or 3+ (positive) by IHC, and classify as amplification (positive, Group 5), equivocal (Group 2,3,4) or negative (Group 1) by ISH. In all of these guidelines, when the HER2 status is negative by IHC and/or ISH, is not indicated the confirmation by an alternate assay. In contrast, the HER2 equivocal cases, by either HER2 IHC or HER2 ISH assays, must be analyzed with an secondary HER2 testing method, or on different tissue blocks with the same testing approach (4, 72). The answer about which of the two methods (IHC or ISH) is better for evaluating the HER2 status, continues to be unknown. Also, with the two latest updates, an important problem was added respecting the 2007 ASCO/CAP guidelines: more HER2 equivocal cases are diagnosed which an increase in reflex HER2 testing (74).

**Table 3:** 2018 ASCO/CAP summary recommendations [original recommendations and focused update recommendations (4)].

	**2013 ASCO/CAP recommendations**	**2018 ASCO/CAP recommendations**
**HER2 IHC CRITERIA**		
Specimens to be tested	All newly diagnosed patients with breast cancer must have a HER2 test performed. Patients who then develop metastatic disease must have a HER2 test performed in a metastatic site, if tissue sample is available.	No change
HER2 score 0 (negative)	No staining is observed or membrane staining that is incomplete and is faint/barely perceptible and within ≤ 10% of tumor cells.	No change
HER2 score 1+ (negative)	Incomplete membrane staining that is faint/barely perceptible and within >10% of tumor cells.	No change
HER2 score 2+ (equivocal)	• Circumferencial membrane staining that is incomplete and/or weak/moderate and within >10% of tumor cells, or • Complete and circumferential membrane staining that is intense and within ≤ 10% of the invasive tumor cells.	• Weak to moderate complete membrane staining observed in >10% of tumor cells. • Basolateral staining for HER2 in a rare subtype of breast cancer with micropapillary histology and circumferential staining that is intense but <10% or the tumor cells.
HER2 score 3+ (positive)	Circumferential membrane staining that is complete, intense, and with >10% of tumor cells that must show homogeneous, darl circumferential (chicken wire).	No change
**HER2 ISH CRITERIA**		
Amplificacion	Dual-probe Her2/CEP17 ratio ≥2.0; with an average Her2 gene copy number ≥4.0 signals/cell (Group 1) Dual-probe Her2/CEP17 ratio <2.0 with an average Her2 gene copy number ≥6.0 signals/cell (Group 3) Dual-probe Her2/CEP17 ratio ≥2.0 with an average Her2 gene copy number <4.0 signals/cell (Group 2)	Dual-probe Her2/CEP17 ratio ≥2.0; with an average Her2 gene copy number ≥4.0 signals/cell (Group 1) Dual-probe Her2/CEP17 ratio <2.0 with an average Her2 gene copy number ≥6.0 signals/cell (Group 3)^†^ Dual-probe Her2/CEP17 ratio ≥2.0 with an average Her2 gene copy number <4.0 signals/cell (Group 2)^†^ If a case has is Group 3 and 2, a definitive diagnosis will be rendered based on additional work-up. If not already assessed by the institution or laboratory performing the ISH test, IHC testing for HER2 should be performed using sections from the same tissue sample used for ISH, and the slides from both ISH and IHC should be reviewed together to guide the selection of areas to score by ISH.
Equivocal	Single-probe average Her2 gene copy ≥4.0 and ≤ 6.0 signals/cell Dual-probe Her2/CEP17 signal ratio of <2.0 with an average Her2 gene copy number ≥4.0 and ≤ 6.0 signals/cell (Group 4).	Dual-probe Her2/CEP17 signal ratio of <2.0 with an average Her2 gene copy number ≥4.0 and ≤ 6.0 signals/cell (Group 4) If a case has an Her2 gene copy ≥4.0 and <6.0 signals/cell ((Group 4)^†^, formerly diagnosed as ISH positive for HER2, a definitive diagnosis will be rendered based on additional work-up. If not already assessed by the institution or laboratory performing the ISH test, IHC testing for HER2 should be performed using sections from the same tissue sample used for ISH, and the slides from both ISH and IHC should be reviewed together to guide the selection of areas to score by ISH.
Non-amplification	Single-probe average Her2 gene copy <4.0 signals/cell Dual-probe Her2/CEP17 signal ratio of <2.0 with an average Her2 gene copy number of <4 signals/cell (Group 5)	No change
**Aceptable (IHC and ISH) tests**	Should preferentially use an FDA-approved IHC, brightfield ISH, or FISH assay	No change

*CAP, College of American Pathologists; CEP17, chromosome enumeration probe 17; ER, estrogen receptor; FDA, US Food and Drug Administration; FISH, fluorescent in situ hybridization; HER2, human epidermal growth factor receptor 2; IHC, immunohistochemistry; ISH, in situ hybridization*.

† *In the 2013 Guideline Update, the work-up of cases in the less common dual-probe ISH categories (groups 2 to 4) include only ISH as additional work-up on diagnosis*.

**Table 4:** Summary of test result scenarios and recommended final HER2 status (4).

**Group**	**Biology**	**HER2/CEP17 ratio**	**HER2 copy number**	**2018 ASCO/CAP recommendation**
1	Classic HER2 amplified cancer^¶^	≥2.0	≥4.0	Positive
2	Monosomy 17^†^	≥2.0	<4.0	Negative, unless concurrent IHC 3+
3	Co-amplification, previously polysomy 17^†^	<2.0	≥6.0	Negative, unless concurrent IHC 2+ or 3+
4	Borderline/equivocal^†^	<2.0	≥4.0 and <6.0	Negative, unless concurrent IHC 3+
5	Classic HER2 non- amplified cancer^¶^	<2.0	<4.0	Negative

¶ *Around 95% of breast tumors tested for HER2 by dual-probe ISH correspond to group 1 (HER2 positive) and group 5 (HER2 negative)*.

† *The overall prevalence of subgroups 2, 3, and 4 among all breast cancers undergoing HER2 testing is estimated to be about 5%, but within and individual laboratory, the frequency ISH results can be increased*.

The concordance between HER2 gene status and HER2 protein expression is generally high, even though discordance between IHC and ISH assay is not uncommon. Both methods detect biological different targets, HER2 protein and HER2 gene expression, respectively, and each assay has its own advantages and disadvantages. The main discordant results are caused by tumor heterogeneity (4, 75–79) focusing mainly in HER2-equivocal cases (4, 73, 76), being a critical factor in the accurate HER2 status evaluation. The ASCO/CAP 2013 guidelines defined heterogeneity as findings of between 5 and 50% of total cells with HER2/CEN17 ratio >2.0 or >6 Her2 signals/cells (72), and the ASCO/CAP 2018 update such as the presence of any aggregated population of amplified cells comprising >10% of the tumor cells on the slide (4). In the low-grade HER2 amplification cases (defined as HER2/CEN17 ratio between 2 and 4) a significant HER2 genetic heterogeneity is detected more frequently than breast cancers with a high-grade HER2 amplification (defined as HER2/CEN17 ratio ≥4.0) and HER2 protein overexpression (defined by IHC 3+) (48, 80). Thus, the evaluation of HER2 through IHC staining and gene amplification, can be remarkably heterogeneous and this could affect the selection of patients, the therapeutic response and the disease-free survival (DFS) rates (76, 81). With an incidence among the studies of 5–40% of HER2 intratumoral heterogeneity (ITH), it cannot be ignored.

HER2 IHC and HER2 ISH tests are employed to select patients for HER2-targeted therapy, and each assay have their advantages and weakness. With the object of improving the assessment of the individual HER2 ITH in tumor samples, Nitta and colleagues, elaborated and validated a protocol in FFPE xenograft tumor tissue sections and in FFPE BC tissue-microarray (TMA) slides, that allows simultaneous brightfield-microscopy detection of HER2 protein and HER2 gene expression, called first *tricolor HER2 gene-protein assay* (GPA) (82). This test exposed the heterogeneity of HER2 protein expression in different BC cells populations (82). A recent study with this assay reported relevant and clinical implications of this intra-heterogeneity (83). Through the combined assessment of HER2 gene amplification and HER2 protein status, five patterns were established. Three of them (type 3 to 5) were defined as a heterogeneous HER2 status and if the tumor case presented any of these types, it related to have ITH. Type 1 (homogeneous HER2 gene amplification and HER2 protein overexpression in all tumor cells) and type 2 (homogeneously amplified HER2 gene tumor cells, but without HER2 protein overexpression) were defined as homogenous HER2 status. The type 1 and type 2 were previously reported as “micro-heterogeneity” (42, 84, 85), what can only be detected by GPA. In the final analyses, the HER2 ITH was an independent factor associated with incomplete pathological response to anti-HER2 neoadjuvant chemotherapy in a cohort of 64 patients (83). Thus, a histopathological-level, a test that allows the recognition of discordance between HER2 gene amplification and protein expression simultaneously, could improve the clinical selection of patients for anti-HER2 therapies, due to a better accuracy of the HER2 IHT in the HER2+ BC.

### Molecular Portraits

HER2+ BC has been historically divided in two distinct diseases based on the expression of hormonal receptors, while the gene expression analyses have proved that HER2+ BC is constituted of all the main intrinsic subtypes. In the HR+/HER2+ BC, two intrinsic subtypes are predominantly isolated: *Luminal B and HER2-E* (43). Within HR–/HER2+ tumors, around 50–88% have the *HER2-E* subtype, followed by other poor prognostic subtypes such as the *luminal B* or the *basal-like* subtype (41). The *HER2-E* subtype is defined by high expression of HER2-related and proliferation-related genes of the 17q amplicon (e.g., ERBB2/HER2 and GRB7), an average expression of luminal-related genes (e.g., ESR1, FGFR4, FOXA1, and PGR) and proteins, and by low or missing expression of basal-related genes and proteins (e.g., cytokeratins 5 and 6, OFXC19) (1, 5). At the DNA level, these tumors are characterized by the greatest number of mutations across the genome. About 70–75% and 40% of HER2-E tumors are TP53 and PIK3CA mutated, respectively (5, 6, 44) (Figure 2). Thus, any HER2+ BC can be included in the *HER2-E, basal-like*, or *luminal* molecular subtypes, and this affect significantly to their biological behavior and therapeutic outcomes. Conversely, the *HER2-E* subtype seems to capture some, but not all clinically HER2+ tumors, while *HER2-E* tumors can be identified within HER2-negative breast tumors, both in hormone receptor-positive or negative profiling (5, 6, 37, 44).

The concept of intrinsic subtypes has provided large insights into the heterogeneity of HER2+ disease. Prat et al. performed an analysis with data of TCGA (5) and METABRIC studies (36) with the purpose to evaluated how molecular subtypes and clinical HER2 status (defined by 2007 ASCO/CAP guidelines and/or DNA copy-number data) overlapped (44). HER2+ BC had a higher frequency of *HER2-E* subtype (47 vs. 7.1% in HER2-negative tumors), with a lower frequency of *luminal A* (10.7 vs. 39%) and *basal-like* subtypes (14.1 vs. 23.4%). Conversely, the ratio of HER2+ BC was 64.6% in *HER2-E* vs. 20, 14.4, and 7.3% in *luminal B, basal-like and luminal A subtypes*, respectively (44). Among HER2+ and HER2-negative BC, <5% genes were found to be expressed differently within each molecular subtype, and respect to the subtype, the genes significant up-regulated in HER2+ breast cancers, were found enriched for genes located in the 17q12 and 17q21 DNA amplicons. The HER2 gene expression and the expression of other 17q12 amplicon genes, were significantly upper in HER2+ tumors with *HER2-E* and *basal-like* intrinsic subtypes. Finally, after a clustering analysis of a METABRIC dataset of the most variable genes across the four subtypes, the results revealed that overall profile of them is largely maintained regardless of the clinical HER2 status, except for the *HER2-E* subtype (44). Thus, it seems that of gene expression the HER2+ BC of a given subtype is practically indistinguishable from a HER2-negative tumor with the identical subtype, except for the higher expression of genes in or close to the HER2 amplicon on 17q in the HER2+ tumors.

In the study about the ten integrative clusters previously described (48), ERBB2 amplified cancers joined in the integrative Cluster 5 (IntClust), unlike the classification of the intrinsic subtypes of Perou et al. (20), or with the analyses of Prat et al. (44). Several publications, have been compared the prognostic value of the 10 integrative clusters classification in front of the intrinsic subtypes, and the authors concluded that they do not confer supplementary information apart from the provided by the intrinsic subtype (44).

The TCGA dataset study also offers the opportunity to examine additional characteristics of the intrinsic subtype based on HER2 status (5). Through the analysis of protein expression, miRNA, DNA methylation and gene expression, slight molecular differences between HER2+ and HER2-negative tumors within each subtype were detected. The vast majority of proteins up-regulated in HER2+ BC derived again from genes located in the 17qDNA region. After the publication of the TCGA study, the last and distinctive study with a similar approach was published in July 2016 (6). The complex molecular heterogeneity within HER2+ disease was highlighted and explained for the first time by whole-sequencing genome (WGS) and transcriptome sequencing data from HER2+ BC samples (6). The authors selected a total of 289 HER2+ breast cancers with FFPE tissues identified within the French PHRE/SIGNAL programs (86, 87). An overall of 99 selected tumors were analyzed for genome-wide expression portraits, out of which 64 tumors and matched normal DNA were subjected to WGS. On the basis of gene-expression data in an unsupervised hierarchical cluster analysis, four groups were defined with specific genomic alterations (somatic mutations, copy-number changes, and structural alterations). Groups A and B encompassed most HR-positive tumors, and groups C and D mostly contained HR-negative tumors. Using the PAM50 assay to identify the intrinsic subtypes, the tumors were mainly *luminal B* (A and B groups) and *HER2E* (in C and D groups), with only a marginal number of luminal A and basal tumors (6). These groups displayed specific genomic alterations too. All samples in group D and none in group A displayed mutations in TP53, while only one sample in group D harbored a mutation in PIK3CA, with equal distribution of such mutations in the other groups. A similar gradient, was also observed in terms of genomic and cell of origin transcriptomic signatures (6, 88). Group D showed more genomic instability and a progenitor luminal signature. In contrast, group A was more stable and showed a typical mature luminal signature (88). These observations are concordant with the cell-of-origin scheme (88–91), in which the intra-tumoral heterogeneity reflects the developmental stage of the epithelial mammary cells. Thus, multiple phenotypes can emerge from one cell-of-origin depending on the initiating genetic event (91).

Thanks to WGS data the authors obtained information about the amplification process itself and about how and maybe when it is arising. The process was consistent with a *breakage-fusion-bridge* (BFB) folding mechanism, supported by the sequence of copy numbers and the orientation of clipped reads (88, 92). However, the present of long distance and inter-chromosomal rearrangements supported that the amplification is a complex phenomenon, probably comprising multiple amplicons on the same or different chromosomes and several interlaced mechanisms (88). All of this suggests that HER2 amplification, although probably strongly selected, is an embedded event that is superimposed on the standard time course of the breast carcinogenesis (88).

Another relevant article recently published, with genomic and transcriptome analysis too, concluded in a similar theory: HER2 could be defined as a pan-cancer phenomenon (93). The authors explored genomics data (RNA sequencing, expression and copy number changes) across three cohorts of patients [TGCA (5), METABRIC (36) consortium and the USO1062 phase III trial population (94)], with more than 3,000 breast tumors samples analyzed. PAM50 was employed for classifying the intrinsic subtypes. Their results were similar to the previously described: (i) the concordance between HER2 amplification and *HER2-E* subtype was really poorly (only 47% of HER2 amplify tumors presented this intrinsic subtype); (ii) it was find no evidence for cooperating copy number drivers with HER2 outside chromosome 17, and finally (iii) after the transcriptional profiling of the *HER2-E* subtype, the authors reported that HER2+ tumors are hormonally driven, either by ER in hormone receptor-positive and *HER2-E* BC, or by AR in hormone receptor-negative and *HER2-E* BC (93).

## Clinical Implications

Trastuzumab was approved in 2001 for metastatic BC patients after the results reported by Slamon et al. (51), in a randomized clinical trial. In adjuvant setting, data from five randomized trials showed a significant improvement in DFS in women with early HER2+ BC after adjuvant treatment with an anti-HER2 antibody called trastuzumab. Latest updates confirmed a benefit sustained over time, resulting finally in a significant improvement in overall survival (OS). In the same way, the treatment with anti-HER2 therapy plus chemotherapy, improved the outcomes in OS in patients with metastatic disease, with numerous randomized clinical trials of anti-HER2 therapies published. To date, the level of expression of HR and HER2 status continue guiding the algorithm of treatment for the HER2+ BC in the clinical practice. Other pathological variables (tumor size, nodal status) provided independent prognostic information. However, if we take into consideration the intrinsic subtype that characterized the tumor, the impact of the clinical and pathological features its decreases considerably.

After the first clinical trial of a HER2-targeted therapy for BC (51), improving strategies to select patients candidate for these therapies has become a critical element to the successful development of anti-HER2 drugs. To date, this selection remaining based on the degree of HER2 positivity in the tumor, by IHC and/or ISH scores (50–53). None biomarker beyond HER2 itself has demonstrated clinical utility across the majority of randomized clinical trials published. Further, although patients with HER2+ disease obtain the greatest benefit from anti-HER2 treatment, the response is greatly heterogeneous, and a substantial proportion of patients present primary or secondary resistance.

The relationship between the grade of HER2 amplification or protein overexpression and the measure of benefit from the different anti-HER2 therapies, has been largely assessed in both early and metastatic disease studies. Available evidence supports a higher probability of success to these therapies in tumors with an increased HER2 protein expression or greater HER2 mRNA levels, although lower HER2 expression or mRNA levels have been associated with clinical benefit too (95–101). Several studies in the neoadjuvant context, have showed an association between rates of pathological complete response (pCR) and a higher HER2 amplification, increased HER2 mRNA levels or HER2 protein overexpression (99–101). In adjuvant studies, such association not impacted either DFS or OS. What's more, centralized laboratory analysis of HER2 testing in the NSABP-B31 (102) and NCCTG N9831 (103) adjuvant trastuzumab trials found a treatment benefit in women with HER2-negative tumors.

Respect to the expression of HR, in the neoadjuvant setting different trials has exhibited heterogeneous response rates after neoadjuvant with chemotherapy and anti-HER2 therapy between hormone receptor-positive and receptor-negative tumors, that is not limited to trastuzumab (10). Achieving a pCR seems to have a significant impact in patient outcomes, with the strongest correlation found in HER2+ BC without expression of hormonal receptors. However, the greatest benefit from anti-HER2 drugs in hormone receptor-negative breast cancers, has not been found in the 3-large adjuvant clinical trials evaluating 1-year of trastuzumab vs. placebo, and both groups seem to obtain similar benefits (103).

Another example that confirm the clinical impact of the HER2 heterogeneity is a phase II study led by the *Danna-Farber Cancer Institute* and recently presented at ASCO 2019 (104). In this clinical trial, the patients received neoadjuvant treatment with 6 cycles of T-DM1 plus pertuzumab. The authors assessment the heterogeneity in basal time (by baseline ultrasound-guide core biopsies from two distinct areas of each tumor), and this entity was defined as at least one of the six areas with either (1) HER2 positivity by ISH in more than 5% and <50% of tumor cells, or (2) a tumoral area with negative result for HER2. Among the 164 patients included, the heterogeneity in HER2 was identify in 10% of evaluable cases without any pCR among cases classified as heterogeneous, being the Residual Cancer Burden (RCB) III the pathological response more frequent in these patients. Secondary analysis also demonstrated a significant relation between pCR (or RCB-0) and HER2 3+ vs. HER2 2+ by IHC. The association between heterogeneity and pCR remained significant when adjusted by hormone receptor status and HER2 IHC measurement (104). These findings, as well as those previously described by Nitta et al. (83), confirm that the ITH is a distinct entity, more diverse than we could expect with the classic pathological evaluation. The heterogeneity in HER2+ BC exist and the treatment of these patients only with anti-HER2 therapies can be insufficient. This entity may need treated with chemotherapy plus anti-HER2 drugs and with novel treatment approaches.

### Sensitivity to Anti-HER2 Based Chemotherapy

The impact of the intrinsic subtyping has been researched retrospectively, either trials evaluating anti-HER2 based chemotherapy in the neoadjuvant [i.e., NeoALTTO (105), CALGB-40601 (84), NOAH (42), CHER-LOB (85) and BERENICE (106)] and adjuvant [i.e., NSABP-B31 (107) and N9831 (108)] settings. Again, in all of these analyses the impact of the HR status, HER2 amplification or the HER2 expression at the protein or mRNA levels, fall into a second or third level such as predictive biomarkers with respect to the intrinsic subtype. In the neoadjuvant setting, when HER2+ BC were clasifficated by PAM50 molecular assay, *HER2-E* subtype was associated with a higher pCR rate (exceeding 50% in all trials) and DFS rates compared to *non-HER2-E* subtypes, following either trastuzumab plus chemotherapy treatment (42, 84, 85) or with dual HER2 blockade without chemotherapy.

### Sensitivity to Dual HER2 Blockade-Only

Nowadays, an area with great interest for the oncologist community is to identify what patients might be treated with a regimen based on dual HER2 blockade without chemotherapy. It has been presented results of several neoadjuvant studies, which submit that a subgroup of patients with HER2+ BC are especially sensitive to the dual HER2 blockade, achieves pCR rates around 70%, so that could potentially be treated without chemotherapy (109).

The *HER2-E* breast tumors are driven by HER2/EGFR signaling, such as it showed, through a silico and omyc analyses, in the TCGA breast cancer project (5). So, this intrinsic subtype should benefit the most from anti-HER2 dual-blockade. The benefit achieved in HER-negative BC with *HER2-E* intrinsic subtype can be explained because these tumors preserve the higher expression of EGFR, with independence of expression degree of hormonal receptors (7). However, the greater response rate in the *HER2-E* subtype in previous studies could not distinguish anti-HER2 sensitivity vs. cytotoxic therapy-sensitivity. *HER2-E* subtype could be a predictor itself of anti-HER2 therapy benefit, and this theory should be validated in future randomized trials. If this happened, this intrinsic subtype could help to select a group of patients with HER2+ BC that might be cured with anti-HER2 drugs without chemotherapy, or patients with metastatic disease that can be treated with less intensive treatment, such as dual HER2 blockade-only.

### Immune Infiltration

The *tumor-infiltrating lymphocytes (TILs)* are white bloodstream cells that migrate toward the tumor. In this heterogeneous group of cells, we have found several types of white cells, including *T cells, B cells*, and even *Natural-Killer* (NK) cells, although the *T cells* are the most representative. Overall, TILs comprising the majority of mononuclear immune infiltrates from the innate and adaptive immune response, with rates that depending of tumor type and stage. An important feature of these cells is that their functions changes dynamically, throughout tumor progression and in response to oncology treatments, being able to acquire dramatically opposite functions. The TILs represent pre-existing anti-tumor immunity, with prognostic relevance and predictive value in BC, especially for HER2+ and triple negative breast cancers (110, 111), although the BC has not classically been considered as an immunogenic neoplasm. In contrast to mucosal tissues, normal breast tissue contain limited aggregates of immune cells (112).

In HER2+ BC patients, the TILS are linked to favorable long-term prognosis and survival outcomes, both on early (110, 113–115) and metastatic disease (116). Within HER2+ BC, non-luminal subtypes have the highest levels of TILs, especially the *HER2-E* intrinsic subtype (7, 117). This has been associated with higher rates of pCR and better survival outcomes following chemotherapy and anti-HER2 neoadjuvant treatment (118), and with response to immunotherapy, as suggest the results from the PANACEA phase IB/II trial (119).

However, in multivariable models adjusted for PAM50 subtypes, TILs seems lost their significant association with better outcomes, due that the intrinsic subtype profiling appears encompasses the information provided by TILs (7). Thus, if immunotherapy aspires to obtain relevance in the treatment of BC, future trials should explore theses new therapies according to the intrinsic subtype, especially in the HER2+ BC.

### Therapeutic Resistance

Different resistance mechanisms to anti-HER2 therapy have been described, which mostly favoring the reactivation of the HER2 pathway or its downstream signaling (109, 120). Most of the therapeutic failures in the treatment of HER2+ BC come from acquired resistance by sub-clones of cells that are highly selected by the therapeutic pressure. The real prevalence and clinical impact of these mechanisms remain largely unclear, majority of them involve genetic or epigenetic aberrations, and have been mainly described in relation to single HER2 blockade (120). Therefore, these mechanisms should clearly be reviewed, because antiHER2 combinations could select different alterations respect to single HER2 blockade.

Among the main mechanisms described we have (1) an incomplete blockade of the HER2 receptor with the activation of compensatory mechanisms by the HER2 receptors family; (2) the activation of alternative receptor tyrosine kinases (RTKs) or other membrane receptors outside of the HER2 family [such as *insulin-like grow factor 1 receptor* (IGF-1R), *AXL Receptor Tyrosine Kinase* (AXL) or *MET* (121)] and (3) the alterations in downstream signaling pathways, especially in the PI3K/AKT/mTOR axis. The hyperactivation of PI3K/AKT/mTOR pathway is the best characterized and seems to be the alteration most important to initiate and perpetuate the resistance to anti-HER2 therapies in HER2+ tumors with any degree of the hormone receptor expression (120). Activating mutations in PIK3CA (122) or reduced levels of tumor suppressor genes (mutations or loss of PTEN, and loss of INPP4-B, among others) are the main molecular alterations than maintain this hyperactivation. The role of targeting these pathways has been evaluated in numerous randomized clinical trials. Among these trials, we have the BOLERO-1 and BOLERO-3, both evaluating the role of everolimus, an mTOR inhibitor, in combination with trastuzumab plus paclitaxel as first-line treatment (BOLERO-1) (63) or in combination with trastuzumab and vinorelbine in trastuzumab-resistant advanced HER2-positive BC (BOLERO 3) (123). The results of them were disappointing and the increase in toxicity very significant. The most relevant data of both studies comes from the combined biomarker analyses that reported an improvement in PFS for patients that harboring PIK3CA mutations or PTEN loss and were treated with everolimus (124). Current efforts have focused on evaluating the activity, in combination with anti-HER2 treatment, of pan-PI3K and alpha-specific PI3K inhibitors. Until now the alpha-specific PI3K inhibitors are the drugs with the most promising therapeutic results and less incidence of serious toxicities respects the pan-PI3K inhibitors (125). These drugs, such as alpelisib (BYL719) (126), target the PI3K-alfa protein, the most frequently altered PI3K isoform in solid tumors and breast cancers, encoded by the PIK3CA gene and with a prominent role in PI3K signaling.

Another relevant mechanism recently proposed is related to the activity of the cyclinD1-cyclin-dependent kinase 4/6 (CDK 4/6) axis. Their enhanced activation can be driven by cyclin D1/CDK4 overexpression or CD4 mutations, causing resistance to hormonal treatment in hormone receptor-positive breast cancers (127). We already have preclinical evidence (128, 129) and from controlled trials (130) about the role of Cyclin D1-CDK 4/6 axis in the anti-HER2 resistance. Using transgenic mouse models, Goel et al. (128) showed that the suppression of CDK4 activity reduces TSC2 (tuberin) phosphorylation, with a partial suppression of mTORC1 and, hence p70-S6K activity, which relieve feedback inhibition of EGFR family kinases rendering cells more sensitive to the effects of EGFR/HER2 inhibitors and overcome acquired resistance to anti-HER2 treatment. The chemotherapy-free trastuzumab-pertuzumab-palbociclib-fulvestrant combination tested in neoadjuvant setting, has recently exhibited promising activity in terms of reduction of ki67 and rate of pCR for breast tumors with positivity of HER2+ and hormonal receptors (130). So, the combination of CKD4/6 and HER2 inhibitors could be a valid option to chemotherapy-containing regimens, at least in a subgroup of patients.

So, hypothetically, the vast majority of resistance mechanisms described could be targeted by drugs that are already available, such as inhibitors of ER, cyclins, mTOR or FGFR1 (109, 120). However, the potential therapeutic advantage of combining these drugs with standard anti-HER2 therapy should be weighed against the potential risk of serious toxicities. Moreover, as a result of intra- and inter-tumoral heterogeneity, different mechanisms can co-exist in a same patient, keeping the potential possibility to contemporaneously target all resistant tumor clones. The *HER2-E* is the second intrinsic subtype, after the *luminal A*, with greater percentage of aberrations in PI3K/AKT/mTOR axis (by PI3KCA mutations/loss of PTEN) and alterations in RB1 pathway (by Cyclin D1 amplification and/or CDK4 gain). So, if both axis are implicated in the resistance of anti-HER2 treatment, this intrinsic subtype could be the most appropriated to design future clinical trials that testing the role of targeting all pathways simultaneously and to prevent of development of acquired resistances, independently of pathological evaluation of HER2, which does not seem to adequately measure the ITH, an entity already established as other potential resistance mechanism.

## Conclusion

To date, amplification and/or overexpression of HER2 remains the only biomarker regarding treatment decisions with anti-HER2 drugs, but it is insufficient itself to clarify the heterogeneous therapeutic outcomes. The complex heterogeneity of the HER2+ BC is a critical aspect, as it has been described at multiple levels: intra-tumoral, at gene expression, transcriptomic and genomic levels. The HER2+ BC do not represent a subtype itself, but are instead dispersed along the whole breast cancer spectrum, from hormone receptor-positive luminal to hormone receptor-negative basal phenotype, with genome variations accordingly to these phenotypes and incidentally defined by a specific gene amplification. Perhaps, combining phenotypic (i.e., gene expression groups) and mechanistic (i.e., co-amplifications) characteristics, may improve the actual classification of HER2+ BC, with the identification of more homogeneous subgroups and improving the knowledge of the genetic mechanisms implicated in the heterogeneity of this disease. This could lead to rational therapeutic strategies, exploring additional pathways and genes co-amplified with ERBB2, especially relevant for patients who show an initial weak response or that exhibit treatment resistance, patients with a particularly poor prognosis.

Although HER2 amplification is traditionally associated with *HER2-E* transcriptional subtype, these are substantially distinct. HER2 amplification seems an oncogenic driver present in all subtypes in place of a biomarker itself of an intrinsic subtype, and its strong enrichment in the *HER2-E* subtype has masked the nature of this entity. Taking into consideration only the intrinsic subtype, any prognostic value attributable to clinical and pathological variables such as the degree, ER/PR or HER2 status by IHC and/or ISH, disappears, as happens with the amplification of HER2 isolated taken as a predictive factor itself.

We already have data of efficacy for anti-HER2 therapy in patients with HER2-negative tumors, with a considerable proportion of patients with HER2+ breast cancers not achieving such clinical benefit. Overall, the evidence so far suggests that all BC with *HER2-E* intrinsic subtype benefit from anti-HER2 treatment. Although much remains to be done, with the data available and presented in this review, it seems that the *HER2-E* intrinsic subtype would be a more appropriate biomarker to assess the real benefit of anti-HER2 treatment in all phenotypes of BC.

Respecting the actual HER2+ BC therapeutic setting, the most recent studies try to improve the results of patients adding new anti-HER2 drugs, still without a selection by molecular features, thus, achieving a discrete therapeutic benefit in the most of these trials, and increasing toxicity and costs. The intrinsic molecular subtyping of BC fairly has extended our knowledge about the behavior of this tumor and should have an established place in the clinical practice. After this revision, we would like to conclude that the *HER2-E* subtype should be established itself as the best predictor of prognosis and clinical outcomes of the BC with this intrinsic subtype, what would allow for the extension of the use of anti-HER2 drugs for HER2-negative tumors and to improve the selection of patients with HER2+ BC for combination of anti-HER2 therapies.

## Author Contributions

AG-O: authorship and complete writing of the manuscript. AS-M and AR: general review of the manuscript and contribution of bibliography. MC: help with the interpretation of molecular data. MP: contribution and bibliographical guidance and has helped too with the interpretation of molecular data. NE: contribution and bibliographical guidance. EC: general review, partial correction of the manuscript, and contribution of bibliography. All authors read and approved the final manuscript.

### Conflict of Interest

The authors declare that the research was conducted in the absence of any commercial or financial relationships that could be construed as a potential conflict of interest.
